# Bright/ARID3A contributes to chromatin accessibility of the immunoglobulin heavy chain enhancer

**DOI:** 10.1186/1476-4598-6-23

**Published:** 2007-03-26

**Authors:** Danjuan Lin, Gregory C Ippolito, Rui-Ting Zong, James Bryant, Janet Koslovsky, Philip Tucker

**Affiliations:** 1 Section of Molecular Genetics and Microbiology and Institute of Cell and Molecular Biology, University of Texas at Austin, Austin, Texas, USA

## Abstract

Bright/ARID3A is a nuclear matrix-associated transcription factor that stimulates immunoglobulin heavy chain (IgH) expression and Cyclin E1/E2F-dependent cell cycle progression. Bright positively activates IgH transcriptional initiation by binding to ATC-rich P sites within nuclear matrix attachment regions (MARs) flanking the IgH intronic enhancer (Eμ). Over-expression of Bright in cultured B cells was shown to correlate with DNase hypersensitivity of Eμ. We report here further efforts to analyze Bright-mediated Eμ enhancer activation within the physiological constraints of chromatin. A system was established in which VH promoter-driven *in vitro *transcription on chromatin- reconstituted templates was responsive to Eμ. Bright assisted in blocking the general repression caused by nucleosome assembly but was incapable of stimulating transcription from prebound nucleosome arrays. *In vitro *transcriptional derepression by Bright was enhanced on templates in which Eμ is flanked by MARs and was inhibited by competition with high affinity Bright binding (P2) sites. DNase hypersensitivity of chromatin-reconstituted Eμ was increased when prepackaged with B cell nuclear extract supplemented with Bright. These results identify Bright as a contributor to accessibility of the IgH enhancer.

## Background

**Figure 1:**
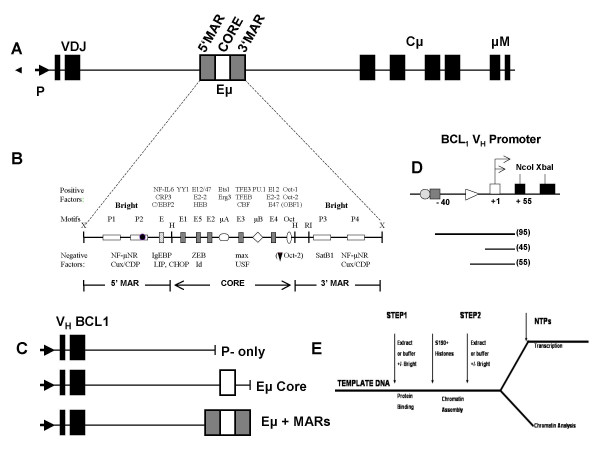
**Schematics of the immunoglobulin heavy chain (IgH) locus and of the templates and strategies used for chromatin reconstituted *in vitro *transcription assays**. **(A)**. Eμ within the rearranged VDJ-Cμ murine (IgH) locus. Promoter, p; direction of transcription, rightward arrow; exons, open boxes; 5' and 3' MARs, hatched boxes; Eμ core, filled box. **(B)**. Detailed schematic of the enhancer sites indicating DNA binding sites and proteins that bind to them to activate (positive factors) or to repress (negative factors) transcription (reviewed in [1]). P1–P4 denote Bright-binding ''P sites'' within the 5' and 3' MARs; the strong P2 site of Bright is indicated by a filled circle. Hinf1 sites (H) operationally define the core region, and XbaI sites (X) flank the MARs. **(C)**. Templates for *in vitro *chromatin assembly/transcription. VDJ and upstream region was derived from the rearranged VDJ expressed by the BCL1 murine leukemia cell line (37). Templates contain VDJ with no enhancer (P-only) or, ~2 kbp downstream, the Eμ core with (+) or without the 5' and 3' MARs. **(D)**. Probes and protected products used for *in vitro *transcription reactions and RNAse protection assays. Transcription *in vitro *and *in vitro *is initiated from two major start sites (indicated by arrows), resulting in protected products (shown below) of 55 and 43 bases. **(E)**. Order of addition and strategy for *in vitro *transcription reactions. As detailed in the text, Step 1 conditions examine transcription following addition of factors (extract only, recombinant Bright, or Bright-supplemented extract) or buffer alone prior to chromatin assembly upon templates. Step 2 conditions measure transcription when factors are added after assembly of chromatin. Transcription is initiated by addition of nucleoside-triphosphates (NTPs).

Numerous studies have demonstrated the requirement of the intronic enhancer (Eμ) in transcription of immunoglobulin heavy chains (reviewed in [1]). *In vivo*, Eμ is required for successful B-cell development, and in its absence, completion of antigen receptor assembly through VDJ recombination is blocked [2,3]. Based on chromatin immunoprecipitation (ChIP) measurements of its histone modification status, Eμ assumes an accessible chromatin configuration specifically in B cells [4-6]. Conventional transcription factors may seize upon this B cell-accessible state to bind to Eμ for transactivation via VDJ-associated promoters (Fig. 1A). Transcriptional activators further exploit increasingly accessible chromatin structures to enhance their binding as B cells progress through development [7].

The Eμ core is flanked on both sides by nuclear matrix associating regions (MARs) (Fig. 1A,B) [8]. As proposed for MARs in general, the Eμ MARs are thought to anchor higher order chromatin into discrete looped domains and to attach them to the nuclear matrix – a site where proteins essential for transcription might reside [9]. While the importance of the Eμ core is universally accepted, the role of their associated MARs remains controversial. The Eμ MARs were initially implicated in locus down-regulation [10-12], an argument strengthened by the observation that the enhancer core alone will activate gene expression in non-B cells [12]. Conversely, the Eμ MARs have been shown to stimulate IgH transcription in B cells (reviewed in [13]), perhaps by impacting chromatin structure of the enhancer [14-17]. For example, targeted *in vivo *deletion of both intronic MARs reduced IgH transcription 5–10 fold [17]. However, deletion of the endogenous MARs in a hybridoma cell line had modest effects, implying a redundant function for the MARs and the core enhancer in maintaining IgH expression [18]. Studies which examined the Eμ MARs in VDJ rearrangement have had variable outcomes, largely depending on the method used to delete the MARs and whether the endogenous locus or a transgenic locus was examined [3,18-20]. A requirement for MAR function *in vivo *but not in cell lines was most convincingly demonstrated by the finding that the Eμ MARs were necessary for generating long-range chromatin accessibility in ectopically integrated reporter gene constructs in transgenic mice [16,17].

Bright, a nuclear matrix-associated, B cell-restricted regulator of IgHtranscription, binds with differential affinity to four ATC-rich motifs (P1–P4, Fig. 1B) within the Eμ MARs to activate transcription of IgH [21]. Bright is stage-specifically expressed in B lymphocytes, where it accumulates primarily within the cytoplasm and the nuclear matrix [22-24]. In addition to its participation in IgH transcription, a function for Bright in cell cycle regulation was suggested by the finding that a fraction of nuclear matrix-associated Bright fractionated into PML nuclear bodies [25]. Consistent with this notion, ectopic over-expression of Bright in embryonic fibroblasts leads to their immortalization via accumulation of Cyclin E and activation of *E2F1 *[26]. Potential relevance of these observations to B-cell malignancy is suggested by the finding that the sub-type of diffuse large B-cell lymphoma with the worst clinical prognosis has elevated levels of Bright [27,28].

Bright is the founder of the 13-member (in humans) ARID (AT-Rich Interaction Domain) family [29]. Bright/ARID3A and several other ARID members (or their fly or yeast orthologues) have been implicated directly or indirectly in chromatin remodeling [30-35]. As often seen with remodeling proteins, Bright has strict contextual requirements for transactivation [21,30]. For example, Bright cannot transactivate via out-of-context, concatenated P binding sites, and transactivation is maximal on integrated substrates [21,30]. Bright binding to its highest affinity P2 site within the Eμ 5' MAR induces severe (80–90)° bending [21,30]. Over-expression of Bright in a mature B cell line induced DNAse I hypersensitivity extending through both Eμ MARs [30]. These results suggest that the enhancer assumes a more open chromatin configuration as a direct or indirect consequence of Bright.

To address the issue directly, we have examined Bright transcriptional activation in an Eμ-responsive chromatin-reconstituted *in vitro *system. Our results support a role for Bright, or a Bright complex which retains Eμ MAR binding, in chromatin remodeling of the enhancer.

## Results

### Rationale and reaction order

*In vitro *transcription on reassembled chromatin templates is the only *in vitro *system in which transcriptional enhancement over distances of 1–2 kb has been achieved (e.g., [36]). Activity requires that the template be packaged into chromatin and that the transcriptional regulatory factors be present before or during chromatin formation so that general repression caused by nucleosome assembly will be blocked.

The 3 template DNAs employed in this study are shown in Fig. 1C, and their construction is detailed in Methods and Materials. Transcription is driven from the promoter of the rearranged VDJ expressed by the BCL1 leukemia B cell line [37]. V_H_BCL1 extends ~270 bp upstream of the 5' most transcriptional initiation site and includes the conserved heptamer and octamer binding motifs [38] (Fig. 1D). V_H_BCL1 has been shown to have strong *in vitro *activity when assayed in nuclear extracts [39,40]. The Eμ core alone (Eμ) or flanked by 5' and 3' MARs (Eμ+MARs) is positioned ~2 kb downstream (or ~400 bp upstream on the circular plasmid backbone) (Fig. 1C).

Our experimental design is shown in Fig. 1E. There are two orders of addition. In the first, naked DNA templates are prebound with nuclear extract from B-cell lines, or with recombinant Bright, or with B-cell extracts supplemented with recombinant Bright (or with buffer) (Step 1). Chromatin is assembled using S-190 extracts from 4 hr *Drosophlia *embryos supplemented with core histones [41,42]. Following chromatin assembly (Step 2), packaged templates are assayed upon addition of nucleotide triphosphates for transcription initiated off of the V_H_BCL1 promoter by quantitative RNase protection. Chromatin alterations are measured by DNase I digestion and indirect end labeling. In the second order of addition, extracts or purified Bright are added following chromatin assemblies.

The Step 1 condition will reveal direct effects on chromatin structure. In this scheme, extracts or purified Bright prebound to the naked DNA template before or during chromatin formation can derepress the general transcriptional repression of assembled nucleosomes. If an effect is seen at addition of extract or Bright at step 2, this would suggest that transcriptional activation requires binding to a pre-formed, reconstituted nucleosome array.

### Assembly of chromatin on IgH templates

**Figure 2:**
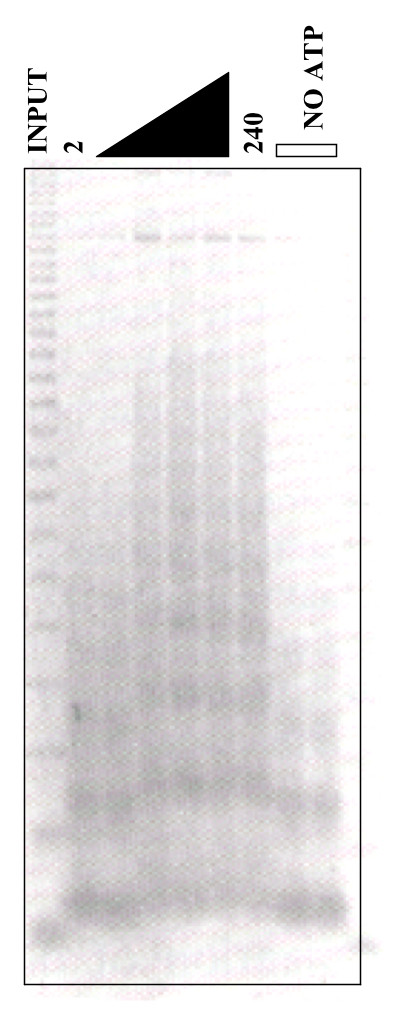
***In vitro *nucleosomal arrays assembled on the IgH enhancer**. The rate and extent of the chromatin reconstitution reaction (detailed in Methods and Materials) on the Eμ+MARs template (Fig. 1C) was monitored by formation of nucleosomes. Aliquots were removed at regular 2–240 min intervals, digested with MNase for 5 min, fractionated on a 1.5% agarose gel, and then stained with ethidium bromide. Outside lanes, naked DNA; -ATP lanes, no ATP regeneration system.

Chromatin assembly on the three templates described above was carried out as detailed in Materials and Methods. A kinetic analysis of micrococcal nuclease (MNase) digestion of assembly on V_H_BCL1-Eμ+MARs is shown in Fig. 2. Assembly was complete in ~30 min, and in the absence of an ATP regenerating system (-ATP lanes), assembly was suppressed. Similar results were obtained for the other templates using this assay and for all templates using a DNA supercoiling assay (data not shown). We conclude that our nucleosomal arrays are sufficient for *in vitro *transcription.

### Transcription from *in vitro *assembled V_H_-promoter-driven templates is responsive to Eμ

**Figure 3:**
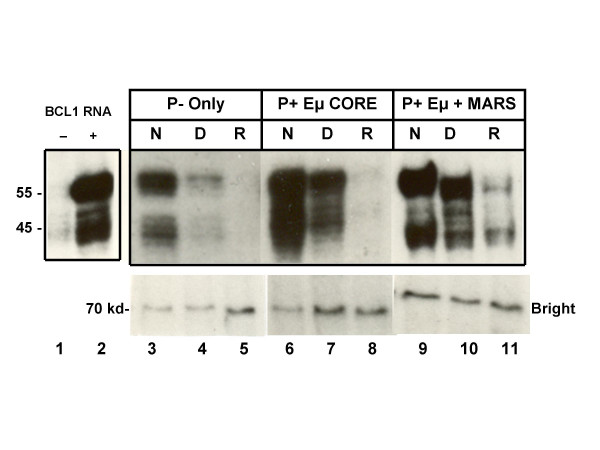
***In vitro *transcription from the V_H_BCL1 promoter is stimulated by the IgH enhancer core alone (Eμ) or the enhancer and associated MARs (Eμ+MARs) on templates reconstituted under Step 1 conditions**. Transcription reactions were carried out as described in Materials and Methods on ~50 ng templates in which either the V_H_BCL1 promoter alone (P-only), or the Eμ core without (Eμ) or with its flanking MARs (Eμ+MARs), were positioned ~2 kbp 3'. Transcription was initiated by addition of NTPs. Upper panel: BCL1 transcripts, which initiate at two major sites *in vivo*, were detected as described in Fig. 1C by RNase protection following fractionation on denatured gels. Lane 1, ~2 μg yeast RNA; lane 2, ~2 μg total RNA from BCL1 leukemia cells; N, naked DNA; D, pre-binding with BJAB nuclear extract (~5 μg/reaction) prior to assembly of chromatin (Step 1 conditions of Fig. 1E); R, post-binding of BJAB extract following assembly of chromatin (Step 2 conditions). Lower panel: Corresponding western blot of SDS-PAGE-fractionated reactions (~15 μg/lane) to monitor and normalize Bright protein levels within input BJAB nuclear extract.

Having succeeded in reconstituting regularly spaced nucleosomes on IgH template DNA, we tested whether the templates of Fig. 1C could direct transcription and, importantly, whether their activities were sensitive to the presence in cis of Eμ or Eμ+MARs. We prepared nuclear extracts from the human Burkitt lymphoma line, BJAB, shown previously by us [39] and others [43] to be highly active for *in vitro *transcription of naked IgH templates. As shown in Fig. 3, transcripts were correctly initiated from all templates in the absence (N lanes) or presence (D and R lanes) of chromatin as confirmed by their sizes relative to authentic BCL1 transcripts (lane 2). Transcription initiated from each template was repressed (R) when reconstituted with chromatin prior to addition of BJAB nuclear extract under Step 2 conditions (R lanes 5, 8, and 11). Importantly, transcription was derepressed (D) for all templates by pre-binding BJAB nuclear extract (D lanes 4, 7, and 10). Equal inputs, confirmed by anti-Bright Western blotting (Fig. 3, lower panel) allowed us to estimate the quantitative effects of *cis*-acting sequences. Importantly, we observed Eμ enhancer-dependent stimulation in this system under Step 1 conditions (compare D lanes ± Eμ ; lanes 4 vs 7). The inclusion of 5' and 3' MARs reproducibly enhanced transcription levels achieved with Eμ alone (compare D lanes, Eμ vs Eμ+MARs; lanes 7 vs 10). We conclude that our chromatin reconstituted *in vitro *transcription system is responsive to the enhancer and adequate to address the central question of the role of Bright. Furthermore, the data are consistent with the conclusions of Forrester et al. [16] in suggesting that Eμ MARs positively contribute to IgH transcriptional activity through a chromatin-based mechanism.

### Choice and production of endogenous and recombinant Bright

**Figure 4:**
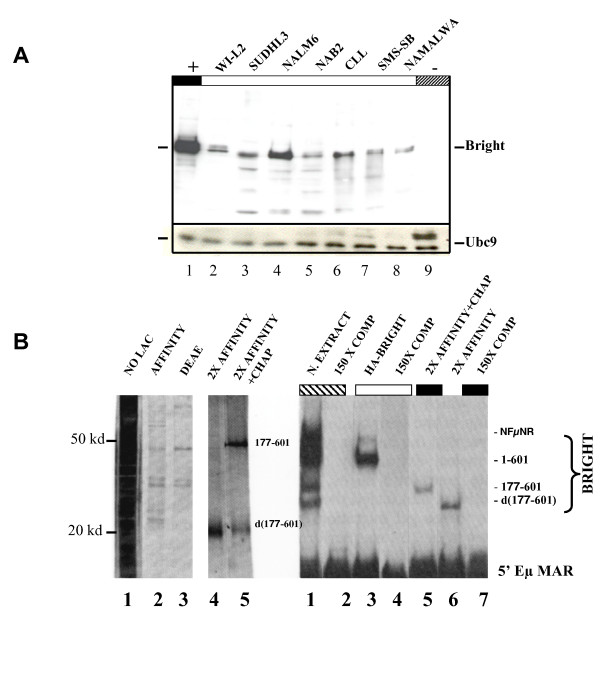
**Preparation of endogenous and recombinant Bright**. **(A) **Bright expression in human B cell lines. Crude nuclear extracts were prepared from the indicated B cell lines (lanes 2–9), from murine embryonic fibroblasts (MEFs) (-, lane 9) or from MEFs transduced with retroviral HA-Bright (+, lane 1). Approximately 15 μg/lane were fractionated on SDS-PAGE, and proteins were identified by western blotting with either an anti-ubc9 mAb (loading control, lower panel) or an affinity purified polyclonal rabbit anti-Bright antibody [21] (upper panel). **(B) **Purification from bacteria and analyses of Lac-inducible, His-tagged Bright (177–601). Left panel: SDS-PAGE/silver stain assay for purification after each step. Affinity chromatography on Ni beads (Affinity, lane 2) was followed by DEAE biogel agarose chromatography (DEAE, lane3). DNA affinity chromatography employing a Sepharose-conjugated, high-affinity Bright binding P2 site trimer [66] produced high yield and purification (not shown). But we were incapable of preventing the protein (grown in DH5α) from degradation to ~20 kD (Affinity 2X, lane 4) unless the plasmid was transformed into K1309 [44], a strain over-producing chaperones *groE *and *groF *(Affinity 2X+Chap, lane 5). Right panel: Specificity of P2 site-containing MAR binding of Bright (177–601) as judged by EMSA/competition. Lane 1, nuclear (N) extract prepared from BJAB B cells; lane 3, HA-Bright prepared from retrovirally transduced MEF nuclear extract; lanes 5 and 6, Bright (177–601) purified from E coli (protein inputs correspond to lanes 5 and 4, respectively, of left panel); lanes 2, 4, and 7, competition (of the corresponding protein sources (indicated by identically colored boxes at top of lanes) with ~150-fold molar excess of a P2 site-containing duplexed oligonucleotide [66]. The endogenous NF-μNR negative regulator [12,66] which binds to the same P sites as Bright (Fig. 1B) is the slower mobility complex indicated in lane 3.

We reasoned that the abundance of endogenous Bright within a B cell nuclear extract would directly correlate with its transcriptional activity. As shown in the Western analysis of Fig. 4A, Bright levels varied broadly among the human B cell lines examined. We prepared standard nuclear extracts fractionated over heparin agarose from the relatively Bright-low (Namalwa, lane 8) and Bright-high (Nalm6, lane 4) cell lines.

Next, we sought to purify recombinant Bright to replace or to complement extracts for reaction Steps 1 and 2. Several methods to produce full-length Bright (1–601) in *E. coli *were attempted, but these attempts were unsuccessful in producing functional protein (data not shown). The material remained insoluble and could not be actively (as judged by EMSA; data not shown) renatured from inclusion bodies. However, we produced sufficient quantities of an N-terminally His-tagged truncation (residues 177–601). This same truncation was previously shown to be indistinguishable from wild-type as an Eμ transactivator in transfected B cell lines [21]. As judged by SDS-PAGE chromatography (Fig. 4B, left panel), Bright (177–601) was purified to near homogeneity by a combination of affinity and ion exchange chromatography. The faster migrating species was confirmed by Western analysis (data not shown) as a Bright degradation product. This degradation was prevented/significantly reduced (Fig. 4B, left panel, lane 5) by transformation into a chaperone over-expressing *E. coli *strain [44].

Following purification, proteins were tested for DNA binding by EMSA. The ~50 kD Bright (177–601) or its ~20 kD (d177–601) degradation product bound to the 5' Eμ MAR-containing P2 site with specificity (Fig 4B, right panel, lanes 5–7) but with apparent lower affinity than endogenous Bright-containing Nalm6 extract (although it was difficult to compare their concentrations quantitatively). Full-length Bright binds to MARs as a tetramer [21], and the C-terminus-proximal REKLES domain (retained in both full-length and 177–601 proteins) is necessary and sufficient for tetramerization (24). Accordingly, the sizes of the DNA-protein complexes were consistent with multimerization of Bright (177–601). We conclude that bacterially-expressed Bright (177–601) is sufficient for use in *in vitro *transcription experiments.

### Bright stimulates *in vitro *transcription by relieving the inhibitory effect of chromatin

**Figure 5:**
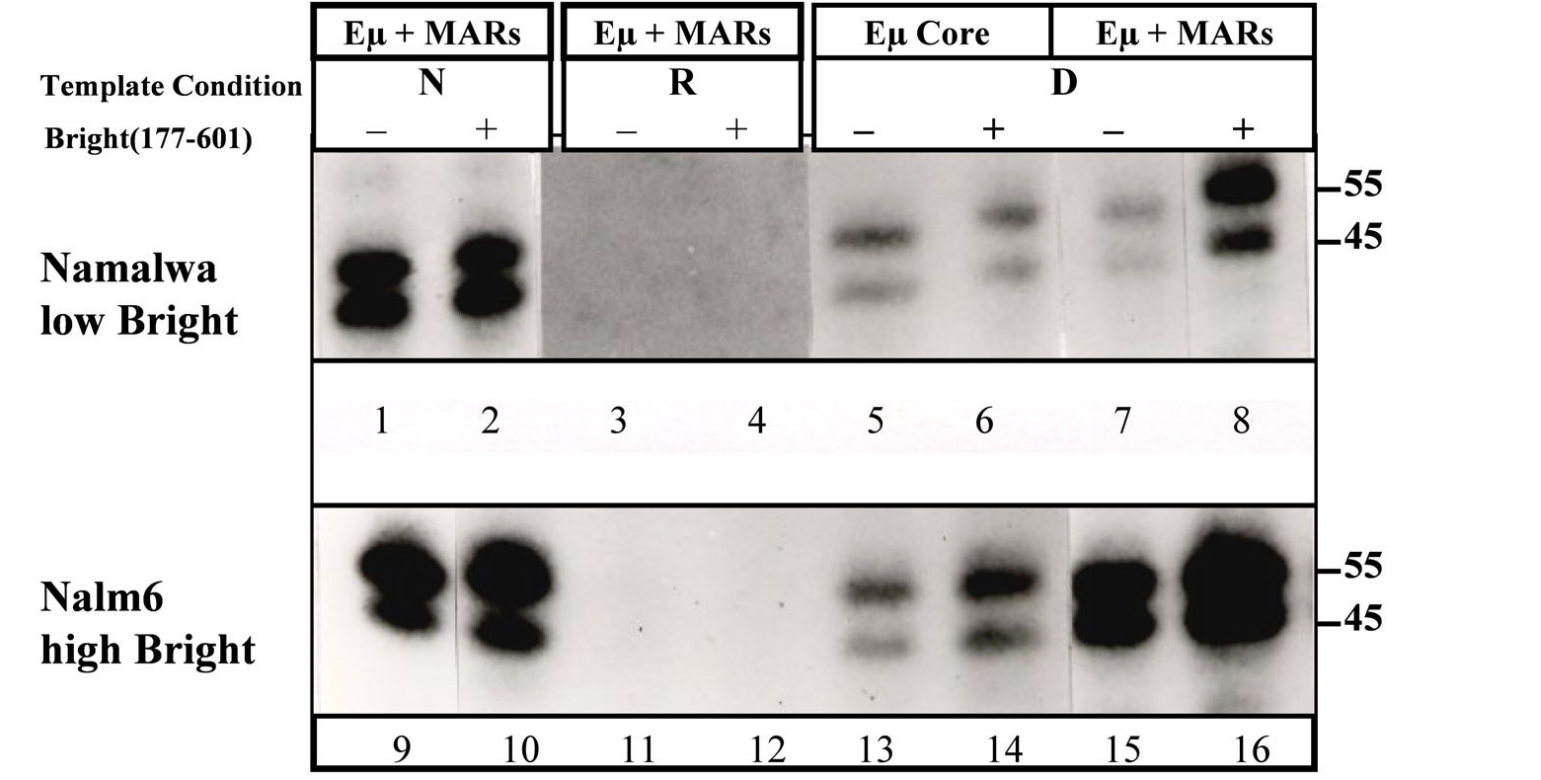
**Levels of *in vitro *transcription from chromatin assembled IgH enhancer templates correlate with levels of endogenous or recombinant-complemented Bright**. Transcription reactions and templates utilizing the Eμ core without (Eμ) or with flanking MARs (Eμ+MARs) were measured by RNase protection as described in legends to Figs. 1, 3, and Materials and Methods. Transcription reactions were performed on N, naked DNA; R, reconstituted chromatin (Step 2 reaction order conditions); or D, prebound chromatin (Step 1 conditions). Protein sources: Upper panel (lanes 1–8): heparin agarose purified nuclear extracts prepared from Namalwa (5 μg/reaction) that contain low levels of endogenous Bright (Fig. 4A, lane 8); Lower panel (lanes 9–16): Nalm6 nuclear extract (5 μg/reaction) containing high levels of endogenous Bright (Fig. 4A, lane 4). Extracts were supplemented in the indicated lanes (+) with ~20 ng of purified Bright (177–601; Fig 4B, lane 5). Shown are phosphoimages of transcription reactions following protein removal and fractionation on 6% acrylamide/5 M urea gels.

Transcription reactions were carried out using the templates, Bright-rich and low-concentration extracts, and recombinant Bright (177–601) protein described above. We summarize all the data in Table 1 and key RNase protection results are presented in Fig. 5. Both nuclear extracts, but not Bright (177–601) alone, stimulated *in vitro *transcription of Eμ and of Eμ+MARs equivalently as naked DNA templates (eg, N lanes 1,2,9,10). These activities were repressed when the factors were added in Step 2 following chromatin-reconstitution on the templates (R lanes 3,4,11,12). Step 1 prebinding of all extracts derepressed (D lanes) both templates to some extent, although consistently stronger activity on the Eμ+MARs template was observed for the Bright-rich Nalm6 extract (compare lanes 13 and 15). Recombinant Bright (177–601) alone was incapable of transactivating any of the templates as naked DNA; nor did addition of Bright (177–601) alone derepress chromatin assembled templates (data not shown; Fig. 5, lane 4; Table 1). However, its addition at Step 1 strongly complemented the ability of Namalwa to derepress Eμ+MARs (compare lanes 7 and 8), while providing a more modest co-activation to Bright-rich Nalm6 (lane 15 vs. lane 16). The data indicate a direct role for Bright in alleviating the chromatin-mediated repression of the enhancer.

**Table 1:** Summary of *in vitro *transcription results of Figures 4, 5 and data not shown.

**EXTRACT**	**BRIGHT (177–601)**	No Eμ			Eμ CORE			Eμ+ MARS		
		N	R	D	N	R	D	N	R	D
**Namalwa**	**-**	+	-	+	++	-	+	++	-	+
**Namalwa**	**+**	+	-	+	++	-	+	++	+/-	+++
**Nalm6**	**-**	+	-	+	++	-	+	++	-	+++
**Nalm6**	**+**	+	-	+	++	-	++	++	+/-	++++
**-**	**+**	-	-	-	-	-	-	-	-	-

Abbreviations are provided in the legend to Fig. 3. Intensity estimates of RPAs are indicated as weak to absent (-) or as increasingly significant (+ to ++++) above the appropriate control for the particular reaction condition step.

### Enhancer derepression by Bright requires P site-specific MAR binding

**Figure 6:**
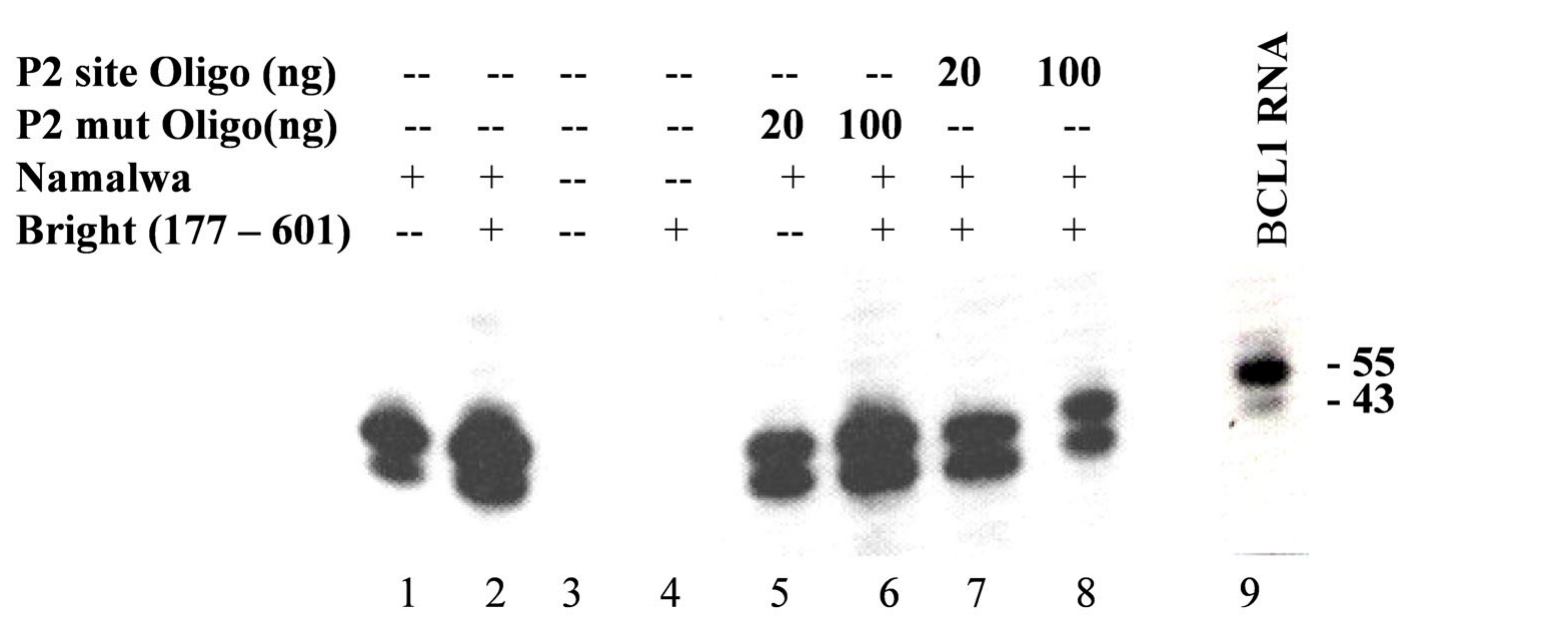
**Competition of Bright-complemented *in vitro *transcription by a high affinity Bright-binding P2 site**. Prior to NTP initiation of *in vitro *transcription on the Eμ+MARs template, either no oligonucleotide (lanes 1,2), or increasing concentrations of a duplexed wild-type P2 oligo (lanes 5,6) or a duplexed mutant (mut) P2 oligo (substituted in 5 core positions to eliminate Bright binding) (lanes 7,8) were added to the reaction prior to chromatin assembly (Step 1 conditions). *In vitro *generated transcripts were measured by RNase protection and compared to total RNA isolated from BCL1 leukemia cells (~0.5 μg; lane 9).

To determine if the Bright (177–601) complementation observed in Fig. 5 required MAR binding, we titrated into Step 1 reactions duplex oligonucleotides corresponding to either a wild-type or mutated 5'- Eμ MAR-containing P2 Bright binding site (Fig. 6) [21,30]. Specific, dose-dependent inhibition of transcription was observed for the wild-type, but not for the mutant P2 oligo. Although there are other interpretations, the simplest is that binding of Bright to its MAR-containing P2 site was specifically competed by excess P2 oligo.

### Bright levels correlate with increased enhancer accessibility

**Figure 7:**
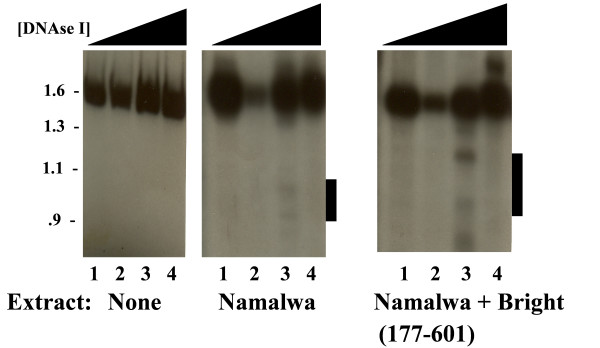
**Complementation of Namalwa with Bright enhances DNase hypersensitivity of chromatin-assembled IgH enhancer**. Following *in vitro *transcription, Eμ+MARs DNA template alone (left panel) or Eμ+MARs reconstituted under Step 1 conditions with either Namalwa nuclear extract alone (middle panel) or Namalwa complemented with Bright (177–601; right panel) were digested with 0, 1.0, 2.0 or 3.0 μg/ml DNase I (lanes 1–4 in each panel). DNA was purified, cut with Bgl II and analyzed by Southern blotting following hybridization with a downstream (XbaI-EcoRI) probe as described in Materials and Methods. Middle and right autoradiographs were exposed ~2.5 times longer so as to better visualize hypersensitive fragments (horizontal bars).

DNase I hypersensitivity sites coincide with nucleosome-free regions in chromatin. We analyzed the accessibility of chromatin assembled *in vitro *on Eμ+MARs to factors present in Namalwa nuclear extract in the absence or presence of Bright (177–601) under Step 1 conditions by DNase digestion (Fig. 7). Preferred sites of DNase I cleavage were examined with respect to a downstream BglI site by using a proximal probe (detailed in Materials and Methods). While the effect was modest, Bright (177–601)-complemented Namalwa extract rendered a consistently increased and extended hypersensitivity across the enhancer (compare lanes 3 among the panels). These results suggest that the enhancer assumes a more open chromatin configuration as a direct consequence of Bright.

## Discussion

Numerous ubiquitous and B cell-specific transcription factors have been identified that transactivate the IgH enhancer (Fig. 1B; reviewed in [1]). Functional analyses underlying most characterizations have relied on transient reporter assays and have ignored to a large extent the physiological role of chromatin. Chromatin imposes an obligatory negative constraint upon enhancer accessibility. Thus, while conclusions derived from reporter approaches are valid in the context of accessible regulatory elements, they do not address many basic mechanisms of enhancer activation.

The concept of locus accessibility is at the heart of antigen receptor VDJ and class switch recombination (reviewed in [45]). However, few bonafide accessibility factors have been identified. Perhaps the best characterized Eμ accessibility factor is the ETS transcription factor family member, PU.1 [46,47]. PU.1 functions through the interaction with another ETS protein, Ets-1, to transactivate Eμ and to stimulate enhancer accessibility in cultured cells via μB site binding (Fig. 1B) [48,49]. Importantly, PU.1 was observed to stimulate *in vitro *transcription and Eμ accessibility from chromatin reconstituted templates [50]. In contrast, another essential Eμ-binding transactivator, E47, appears to function indirectly by weak binding to accessible μE5/μE2 sites (Fig. 1B) [51,52].

We previously showed that Bright/ARID3A, when over-expressed in cultured WEHI 231 B cells, facilitated DNase I hypersensitivity of Eμ [30]. Four other members of the 13 member ARID family (including SWI1/p270 of SWI/SNF) have been directly or indirectly implicated in chromatin remodeling [31-35]. Prompted by these observations, we established a system in which transcription from *in vitro *assembled V_H_-promoter-driven templates was responsive to Eμ. We found that Bright could complement other B cell-derived factors to derepress the inhibitory effects of chromatin assembled on the enhancer. The Eμ flanking MARs were required for maximal Bright-mediated *in vitro *transactivation. We demonstrated that the DNase I hypersensitivity of chromatin assembled *in vitro *on the enhancer was increased by Bright. These data indicate a direct role for Bright and further support a role for the Eμ MARs in facilitating a fully accessible chromatin state of Eμ.

Our previous analysis [30] and unpublished MNase digestion experiments on isolated B cell nuclei suggested that Bright may function by Eμ nucleosomal disruption. The simplest mechanism to explain this effect would require that Bright reach the enhancer in the context of heterochromatin. However, the results reported here showed that Bright could alleviate chromatin-mediated repression only if it was delivered prior to chromatin assembly. That is, Bright cannot activate *in vitro *transcription by binding to a preformed nucleosome array. In contrast, *in vitro *assembled chromatin footprinting experiments revealed that PU.1 is capable of binding μB in the repressive context of chromatin [50]. The authors speculated that PU.1 might provide a platform for assembly of a "targesome", a protein complex required for a fully accessible chromatin structure [53]. Bright might participate in such a complex. However, as with PU.1 [50], our competition experiments indicated that Bright required an intact DNA binding site to mediate maximal Eμ chromatin accessibility. This suggests that Bright is recruited independently and perhaps subsequently to PU.1, through direct binding to its P site(s). Both PU.1 and Bright might function to clear out nucleosomes otherwise positioned over critical cis-acting regulatory elements within the Eμ core to provide accessibility to conventional DNA-binding transactivators.

Regulation of chromatin structure by conventional protein-DNA interactions is generally considered to act only proximal to the DNA binding site [54,55]. MARs might offer an exception to this case. Forrester et al [16,56] demonstrated that the Eμ MARs were required to obtain normal transcription initiation rates and to produce extended DNase I hypersensitivity across a VDJ-associated promoter over 2 kbp away. The mechanism underlying such distal accessibility induction is unknown, but it seems reasonable to speculate that a MAR-binding accessibility factor might contribute. As mentioned in the Background section, the contradictory evidence on Eμ MAR function rests to a large extent on whether the endogenous locus or a transgenic locus was investigated [3,18-20,34-36,57]. For example, studies using chimeric mice with targeted deletion of the Eμ MARs reported that these elements were dispensable for VDJ recombination and transcription of the endogenous IgH locus [3]. However, while the endogenous and MAR-deleted alleles were expressed at similar levels in splenic IgM^+ ^B cells [3], the total numbers of IgM^+ ^B cells in mice with a MAR deletion were less than half of those observed in wild-type mice or mice with deletion of only the Eμ core. This suggests that deletions of the MAR elements may result in defects in B-cell development that have yet to be fully appreciated. The requirement for MAR function in transgenic animals, but not in cell lines or animals created from blastocyst fusions, is consistent with a MAR function in chromatin remodeling during early development or passage through the germline. This is consistent with the results of Forrester et al. [16] and those presented here.

In addition to Eμ, IgH-associated MARs often reside 5' of V_H _promoters [58-60]. A MAR upstream of the S107 variable region V_H_1 promoter was shown to contain specific Bright-binding P sites [59]. Indeed, Webb and colleagues have convincingly demonstrated that Bright can associate with both Bruton's tyrosine kinase and TFII-I to activate transcription of a S107 V_H_1 reporter through this proximal MAR in the absence of Eμ [61,62]. The existence of V_H _and Eμ-associated MARs and the ability of Bright to form multimeric MAR binding complexes [21] offers the possibility of looping enhancers and promoters into close proximity to stimulate transcription through nuclear matrix attachment-mediated domain formation [23]. Whether the *in vivo *mechanisms underlying promoter-proximal (V_H_) and promoter-distal (Eμ) MAR-mediated transactivation by Bright are the same and can be accommodated by the looping model remain to be tested. In this context, we note that Bright levels in adult mice spike distinctly in large preB and mature B cells [22]. At the latter stage, maximal Bright expression and V_H_1 DNA binding are induced by mitogens and cytokines (e.g., LPS, IL-5, CD40L) that drive B lymphocytes into the cell cycle [21,22]. Perhaps Bright might utilize quite different transactivation options and/or function through different IgH MAR-associated binding sites under circumstances in which accessibility of Eμ has already been established.

Finally, we suggest that Bright may contribute to chromatin remodeling at loci other than IgH. Bright was shown to rescue primary fibroblasts from natural replicative senescence or from premature senescence induced by oncogenic RAS^V12 ^[26]. As with several other ARID factors [31,63], Bright binds retinoblastoma protein (Rb) (C. Schmidt and PWT, unpublished results), leading to the possibility that this tumour suppressor pathway is inactivated during senescence rescue. This hypothesis is consistent with the observation that Bright over-expression in MEFs activates E2F1 and Cyclin E1 [26]. Dean and colleagues [64] have provided a chromatin-based explanation for Rb/E2F transcriptional regulation which could accommodate a contributor with the properties of Bright.

## Conclusion

We established a chromatin-reconstituted, *in vitro *transcription system which is responsive to the IgH enhancer. Our results support the conclusion that Bright contributes to enhancer function by increasing its accessibility through matrix attachment site binding.

## Materials and methods

### Constructs, probes and oligonucleotides

The template plasmids for *in vitro *transcription were constructed by cloning the 593 bp BamH1-XbaI fragment that spans the rearranged VDJ expressed by the BCL1 leukemia cell line [37] into pUC19. This fragment (V_H_BCL1) contains ~270 bp upstream of the 5' most transcriptional initiation site, including the conserved heptamer and octamer binding motifs [38]. V_H_BCL1- Eμ was constructed by inserting the Eμ enhancer core, as a 220 bp HinfI fragment, ~2 kbp downstream in transcriptional sense (~400 bp upstream on circular plasmid) of V_H_BCL1. V_H_BCL1-Eμ+MARs was constructed by inserting Eμ along with its flanking 5' and 3'MARs as a 911 bp Xba I fragment into the same location relative to V_H_BCL1. For the V_H_BCL1 antisense RNase protection probe, a 322 bp BamH1-NruI fragment containing ~55 bp downstream from the major initiation of transcription site was cloned into pGEM4 to generate pBCL1-5'. The plasmid was linearized with HinfI and transcribed *in vitro *by sp6 polymerase (Promega) to generate a 95 b RNA probe. Oligonucleotides corresponding to the + and - strands of wild-type (5'-CTTTTAACAATAATAAATTAAGTTTAAAATATTTTT-3') or mutated (underlined bases changed to TAATT) P2 Bright binding site within the Eμ 5' MAR were synthesized, annealed, and the resulting duplex was gel purified as previously described [21].

### Cells

The BCL1 murine leukemia, and human Burkitt's lymphomas (BJAB, Nalm6 and Namalwa) were maintained in RPMI supplemented with 10% fetal calf serum. For protein purification, we employed either *E. coli *BL21 Star (Invitrogen) or E coli K1309 [44], a strain overproducing chaperones *groE *and *groF *kindly provided by Dr. G. Georgiou (UT Austin, Dept. of Chem. Engineering). Induction of the chaperones was induced with 10 ng/ml^-1 ^tetracycline at the beginning of the incubation in LB or M9 media.

### RNase protection

Labeling, purification, and denaturating gel analysis of the pBCL1-5' riboprobe were carried out as previously described [39]. After hybridization at 60°C overnight, unduplexed probe was digested with 40 ug RNase A (Sigma)/ml and 2500 U RNase T1 (BRL) for 1 hr at 37°C. Protected RNA fragments were separated on 6% acrylamide gels containing 8 M urea, and autoradiography was carried out for 24–96 hr.

### Protein manipulations

Nuclear extracts were prepared by the method of Dignam et al. [65] with minor modifications as described by Johnson et al. [39] to achieve final protein concentrations of 8–10 mg/ml.

An N-terminal 6X-histidine-tagged Bright truncation (amino acids 177–601) was constructed as previously described [21], cloned into the pET30a+ expression vector (Novagen), and its expression induced with IPTG 30 min after chaperone induction (see above). Harvested cells were disrupted by sonication, and total cell lysates were analyzed on 12% SDS-PAGE (prior to or as a monitor of purification) with SilverStain (Invitrogen). Following elimination of cell debris by centrifugation, supernatants were purified by affinity chromatography over Ni2^+^-NTA agarose SuperFlow according to the manufacturer's instructions (Novagen and Qiagen). Further purification was carried out by DEAE Bio-Gel agarose chromatography as instructed by the vender (Pharmacia Fine Chemicals). The Bright-containing fraction was subjected to DNA affinity chromatography employing a Sepharose 6B-conjugated, high affinity Bright binding P2 site trimer (synthesis and elution conditions as described [66]). The final yield of purified Bright (177–601) was 8–20 μg from 2 l of M9 or LB media, respectively.

Western analysis was performed according to Kim and Tucker [24]. Proteins were separated by 9% SDS-PAGE and transferred to Protran nitrocellulose membranes (Perkin Elmer). The membranes were incubated with anti-Bright polyclonal [21] and then developed with goat anti-rabbit IgG peroxidase-conjugated secondary (Amersham). Bands were visualized with ECL Western Blotting Detection Reagents (Amersham).

### Electrophoretic mobility shift assays (EMSA)

*In vitro *DNA binding and antibody supershift reactions were performed as previously described [21,66]. Briefly, either ~2 μg of nuclear extract or ~20 ng of purified Bright (177–601) was incubated with ~80,000 cpm of 5' end-labeled and gel-purified Bright-specific 5' Eμ MAR probe (Fig. 1B). Samples were incubated for 20 min at room temperature and then resolved on 4% polyacrylamide gels.

### Chromatin reconstitution and *in vitro *transcription

Chromatin was assembled onto circular plasmid DNA templates using *Drosophila *core histones and S-190 assembly extract, derived from *Drosophila *embryos as previously described [41,42]. Briefly, template DNA (~500 ng), core histones (~400 ng), S-190 (~3.0 μg), 3 mM ATP plus an ATP regenerating system (30 mM phosphocreatine and 1 μg phosphocreatine kinase/ml) were incubated in 60 mM KCl. To monitor assembly, aliquots (~100 ng DNA) were removed at regular 2–240 min intervals, and then digested with micrococcal nuclease (MNase) (0.4 units/ml) for 5 min in a 30 mM CaCl_2_-containing, 10 mM Hepes (pH 7.5) buffer supplemented as previously described [42]. Reactions were deproteinized by protein K digestion, extracted with phenol/chloroform, ethanol precipitated, fractionated on a 1.5% agarose gel, and then visualized by ethidium bromide staining. Optimal assembly was achieved at ~1:.7 ratio of core histones: DNA.

*In vitro *transcription was carried out as described [41,42] on ~50 ng naked or chromatin reassembled V_H_BCL1, V_H_BCL1-Eμ or V_H_BCL1-Eμ+MARs templates. Transcription was initiated by addition of 10 mM nucleoside triphosphates (NTPs). Complementation experiments were carried out by addition of B cell nuclear extracts (~5 μg/reaction) and/or purified Bright (177–601) (~20 ng/reaction). Variable orders of reaction were described in Results.

### Chromatin accessibility measurements

DNase I digestion analysis of reconstituted chromatin templates was performed as described [30]. Aliquots (~100 ng DNA) were digested with DNase I (Worthington; 75 μg/ml) for various times at room temperature, purified by proteinase K digestion, phenol/chloroform/isoamyl alcohol (25:24:1) extraction and ethanol precipitation. Digests were restricted with *Bgl*II, fractionated on a 1.4% agarose gel containing 40 mM Tris acetate and 1 mM EDTA. The DNA was blotted overnight (Bio-Rad Zeta probe), neutralized, baked under *vacuo*, and then prehybridized for 2 hr overnight in 0.3 M NaCl, 15 mM sodium phosphate (pH 7.0), 1.5 mM EDTA, 0.5% BLOTTO dried milk powder, 1% SDS, and 500 μg/ml sonicated herring testis DNA. The blot was hybridized overnight in the same buffer to an ~300 bp *Xba*I-*Eco*RI restriction fragment (downstream to the Eμ 3' MAR; Fig. 1B) radiolabeled to a specific activity of ~10^9 ^cpm/μg (~2.5 × 10^7 ^cpm) with a DNA labeling kit (Ambion). The sizes of hypersensitive fragments were estimated from linear fit of log DNA size vs mobility relative to DNA standards.

## Competing interests

The author(s) declare that they have no competing interests.

## Authors' contributions

DL, GCI, RTZ and JB performed in vitro transcription experiments, prepared and analyzed data, prepared figures and contributed to manuscript drafts. JK carried out protein purifications. PT directed the project, finalized the manuscript and procured funds to support the work.
